# Revealing the role of a novel *IDS* gene mutation in mucpolysaccharidosis type II: insights from computational analysis

**DOI:** 10.3389/fmolb.2026.1734111

**Published:** 2026-04-02

**Authors:** Shanzhou Huang, Chonghan Li, Yuyuan Zhi, Zengling Su, Fei Ma, Congcong Shi, Sitao Li

**Affiliations:** 1 Department of Neonatology, Yuexi Hospital of The Sixth Affiliated Hospital, Sun Yat-sen University/Xinyi People’s Hospital, Xinyi Maoming, Guangdong, China; 2 Department of Rehabilitation Medicine, Yuexi Hospital of The Sixth Affiliated Hospital, Sun Yat-sen University/Xinyi People’s Hospital, Xinyi Maoming, Guangdong, China; 3 Maternal & Child Health Research Institute, Zhuhai Center for Maternal and Child Health Care, Zhuhai, China; 4 Department of Pediatrics, The Sixth Affiliated Hospital, Sun Yat-sen University, Guangzhou, Guangdong, China; 5 Biomedical Innovation Center, The Sixth Affiliated Hospital, Sun Yat-sen University, Guangzhou, Guangdong, China

**Keywords:** frameshift mutation, genome-scale metabolic analysis, iduronate-2-sulfatase, mucopolysaccharidosis type II, protein structural analysis

## Abstract

**Introduction:**

Mucopolysaccharidosis type II (MPS II; Hunter syndrome) is an X-linked lysosomal storage disorder caused by variants in the *IDS* gene. This study reports a male infant with a novel hemizygous frameshift mutation (*IDS* gene: NM_000202.8, c.1133delA, p.Phe378SerfsTer13). We will investigate the functional consequences and pathogenic mechanisms of this novel mutation.

**Methods:**

The mutation c.1133delA in the *IDS* gene of this patient was confirmed by Sanger sequencing. Structural modeling was performed to assess the impact of the mutation on protein architecture. Additionally, a genome-scale metabolic model was employed to simulate the metabolic consequences of IDS deficiency.

**Results:**

Structural analysis revealed deletion of the sulfatase domain 2 (SD2) and disruption of the ligand-binding pocket. Metabolic modeling demonstrated that perturbations were highly localized, affecting only a limited subset of reactions primarily confined to glycosaminoglycan degradation pathways, without detectable impact on core cellular metabolism. The model further predicted accumulation of glycosaminoglycan-related intermediates, consistent with known biochemical hallmarks and clinical manifestations of MPS II.

**Discussion:**

This study demonstrates the pathogenicity of the mutation c.1133delA, our findings highlight the value of metabolic network analysis in understanding disease mechanisms and identifying potential therapeutic targets for MPS II.

## Graphical Abstract

**Figure:**
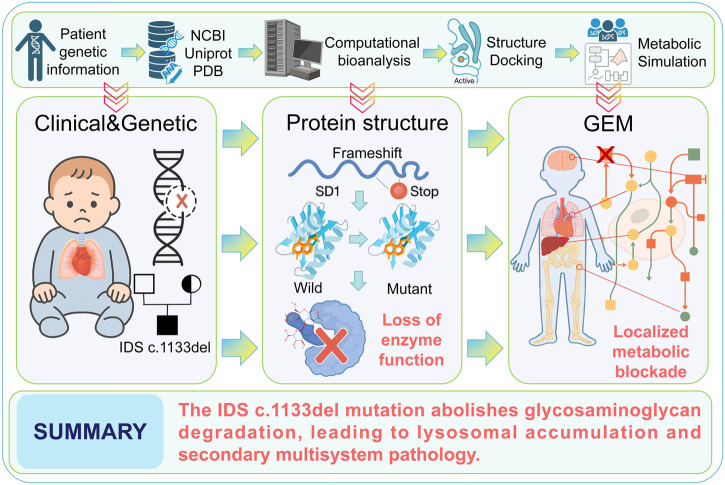

## 1 Introduction

Iduronate-2-sulfatase (IDS) is an important enzyme involved in the degradation and recycling of complex glycosaminoglycans (GAGs) in humans. IDS catalyzes the C2-sulfate bond hydrolysis at the non-reducing end of 2-O-sulfo-α-L-iduronic acid residues in dermatan sulfate and heparan sulfate. Loss of IDS activity results in the abnormal accumulation of GAGs in multiple tissues and organs, leading to progressive cellular and multi-organ dysfunction, thereby causing Mucopolysaccharidosis Type II (MPS II, OMIM 309900) (Neufeld and Muenzer, 2019), also known as Hunter syndrome. Generally, MPS II progression follows two patterns: The “severe” early-onset form, in which symptoms become apparent at 2–4 years of age, presenting behavioral disorders, progressive intellectual impairment with neurodegeneration, and death occurring before adulthood; the other is the “mild” late-onset form, in which patients maintain normal or mildly impaired intelligence and can live into late adulthood. Expression studies have found that some mutations in the *IDS* gene, primarily missense mutations, significantly affect enzyme function or stability. Cases have indicated that patients with severe structural changes in the *IDS* gene due to gene deletion or gene-pseudogene recombination usually present with severe mucopolysaccharidosis (Froissart et al., 2007). Beyond impaired glycosaminoglycan degradation, lysosomal enzyme deficiencies may induce system-wide metabolic perturbations, contributing to the phenotypic heterogeneity observed in MPS II.

IDS deficiency is not limited to lysosomal substrate accumulation but may perturb interconnected metabolic pathways through secondary adaptations. Therefore, network-level modeling provides an appropriate framework to propagate local enzymatic defects into global metabolic responses and facilitate mechanistic interpretation of genotype–phenotype relationships. Genome-scale metabolic models (GSMMs) provide a powerful framework for systematically analyzing the metabolic consequences of genetic perturbations at the systems level. Similar integrative computational strategies have been successfully applied in other disease contexts, where gene expression profiles were combined with molecular interaction prediction to identify therapeutic vulnerabilities and repurposed drug candidates. For example, Nayak et al. demonstrated that integrating gene-level perturbations with molecular interaction analyses through multi-scale computational modeling can generate testable therapeutic hypotheses, such as the identification of BMS345541 in temozolomide-resistant glioblastoma (Nayak and Mallick, 2024). Following the development of Recon1, successive refinements including Recon3D and Human-GEM have substantially expanded the scope and accuracy of human metabolic network modeling (Rolfsson et al., 2011; Brunk et al., 2018; Robinson et al., 2020).

Computational biology approaches were applied to investigate the structural changes and functional impacts of the novel *IDS* gene frameshift mutation c.1133delA, (p.Phe378SerfsTer13) in the IDS protein. To explore whether IDS deficiency induces metabolic perturbations beyond glycosaminoglycan degradation, we employed the human genome-scale metabolic model Human-GEM (Robinson et al., 2020) using the COBRA Toolbox (Heirendt et al., 2019). Flux variability analysis (FVA) was applied to systematically characterize the metabolic consequences of *IDS* knockout. In this study, we applied integrated structural analysis, molecular docking, and genome-scale metabolic modeling to investigate the potential pathogenic mechanisms of a novel IDS frameshift mutation. Additionally, by examining the effects of amino acid changes, domain function analysis, and genome-wide metabolic analysis, this study attempts to explain how this mutation contributes to disease development.

## 2 Methods

### 2.1 Genetic testing

On the second day after birth, the patient underwent newborn genetic screening. The screening panel comprised a total of 160 genes and was conducted using high-throughput sequencing technology. Sequence alignment and variant analysis were performed using the human reference genome (GRCh37/hg19). The interpretation of sequence variants was based on the “Standards and guidelines for the interpretation of sequence variants” of 2015, developed by the American College of Medical Genetics and Genomics (Richards et al., 2015). Positive variants identified in genetic screening are validated by Sanger sequencing. After obtaining informed consent from the patient’s parents, peripheral blood specimens were collected from both the patient and his parents for Sanger sequencing. DNA extraction (DNA extraction kit; Tianlong Science and Technology Co., Ltd., Xi’an; Shaanxi Medical Equipment Registration No. 20140007), genetic screening (Illumina NextSeq 500 × 550 platform; PE150) and Sanger sequencing (ABI sequencing platform) were all conducted by Guangzhou Sheng’an Medical Laboratory Co., Ltd. This study was conducted in accordance with the principles of the Declaration of Helsinki, and was approved by the Ethics Committee of Yuexi Hospital of The Sixth Affiliated Hospital, Sun Yat-sen University/Xinyi People’s Hospital (Ethics committee batch number 2024XYPHEC-024).

### 2.2 Protein structure and functional computational analysis

The protein sequence of IDS was obtained from UniProt (https://www.uniprot.org/uniprotkb/P22304). The X–ray resolved protein structure 5FQL.pdb (Demydchuk et al., 2017) from the PDB database (Rose et al., 2011) was used as the template structure. Using the SWISS-MODEL (Waterhouse et al., 2018) homology modeling method, models of the wild-type and mutant IDS proteins were constructed to obtain accurate mutant structure models. The ligand of the protein, dermatan sulfate, was obtained from the PubChem database (https://pubchem.ncbi.nlm.nih.gov/compound/32756). To evaluate the pathogenic potential of the frameshift mutation, MutPred-LOF, a specialized extension of the MutPred framework designed for loss-of-function variants, including frameshift and stopgain mutations, was employed (Pejaver et al., 2020). Because the mutation leads to premature termination of protein transcription, with part of the truncated region located near the ligand-binding pocket, AutoDock4 (Morris et al., 2009) was used to dock the ligand molecule, dermatan sulfate, onto the wild-type and mutant protein structures, and the interactions of the protein-ligand complexes before and after the mutation were analyzed using the Protein-Ligand Interaction Profiler (PLIP) (Adasme et al., 2021) online server. Hydrogen bonding, hydrophobic interactions, and salt bridges were compared between wild-type and mutant complexes at the residue level. Protein structures were visualized and annotated using PyMOL (Lill and Danielson, 2011).

### 2.3 Human genome-scale metabolic model analysis

Genome-scale metabolic modeling was performed using Human-GEM version 1.19.0 (Robinson et al., 2020), a comprehensive reconstruction of human metabolism comprising 12,971 reactions, 8,455 metabolites, and 2,887 genes. All computational analyses were performed using the COBRA Toolbox (Heirendt et al., 2019) in MATLAB R2024b, with Gurobi 12.0.1 as the linear programming solver.

#### 2.3.1 *IDS* gene knockout simulation

Based on gene–protein–reaction (GPR) associations in Human-GEM, the *IDS* gene (Ensembl ID: ENSG00000010404) is linked to three metabolic reactions: MAR07517 (dermatan sulfate desulfation), MAR07241 and MAR07247 (heparan sulfate desulfation). *IDS* deficiency was simulated by constraining the flux bounds of all three reactions to zero, thereby mimicking complete loss of *IDS* activity. The wild-type model retained original reaction constraints. In all simulations, the default biomass reaction (MAR13082) was used as the objective function.

#### 2.3.2 Flux balance analysis (FBA)

Flux balance analysis (FBA) was performed to assess whether *IDS* deficiency affects cellular growth capacity (Orth et al., 2010). Maximum biomass flux was computed for both wild-type and *IDS*-deficient models under identical constraints. No further flux-based interpretation was derived from FBA results.

#### 2.3.3 Flux variability analysis (FVA)

Flux variability analysis (FVA) was employed to systematically assess metabolic perturbations induced by *IDS* knockout (Mahadevan and Schilling, 2003). Unlike FBA, which yields a single optimal solution, FVA computes the minimum and maximum allowable flux for each reaction while maintaining a specified fraction of optimal growth, thereby accounting for alternative optimal solutions.

Based on preliminary analysis, a 90% optimality threshold was selected to represent a physiologically relevant near-optimal growth state. Sensitivity analyses were further performed at 75% and 50% optimality thresholds to assess robustness.

For each reaction, changes in flux range between wild-type and knockout models were quantified as:

$$\Delta range\left.=\right|\max_{KO}-\max_{WT}\;\left|+\right|\min_{KO}-\min_{WT}\left.\;\right|$$

The distribution of Δrange values exhibited a clear bimodal pattern, and |Δrange| > 1 was selected as the significance threshold.

#### 2.3.4 Sensitivity analysis

To evaluate the robustness of the findings, FVA was repeated under 75% and 50% optimality constraints. The number and subsystem distribution of affected reactions were compared across thresholds.

#### 2.3.5 Reporter metabolite analysis

Reporter metabolite analysis was performed to identify metabolites potentially accumulating due to *IDS* deficiency. Metabolites were considered potentially affected if all consuming reactions were active in the wild-type model but fully blocked in the *IDS* knockout model.

## 3 Results

### 3.1 Clinical manifestation

The patient was a male infant, the first child of a primigravida, born at 40 weeks and 5 days of gestation. During delivery, fetal heart rate irregularities were observed, and the umbilical cord was wrapped once around the neck, with placental calcification. At birth, an accessory ear was noted on the left side, with noticeable Mongolian spots on the limbs and buttocks.

At 5 days of age, the infant was found to have patent ductus arteriosus, a patent foramen ovale, mild tricuspid regurgitation, and mild pulmonary hypertension, along with pathological jaundice, neonatal hyponatremia, and scalp edema.

At 4 months of age, visual and auditory examinations were normal. Muscle tone examination indicated abnormal incoordination. In terms of development, the child could lift the head at 3 months, sit independently at 6 months, and crawl at 8 months, with no signs of developmental delay or intellectual disability. Enzyme activity analysis revealed that the activity of IDS in the patient’s sample was less than 1.00 nmol/4 h/mg of protein (The normal range is greater than 1.00 nmol/4 h/mg).

**FIGURE 1:**
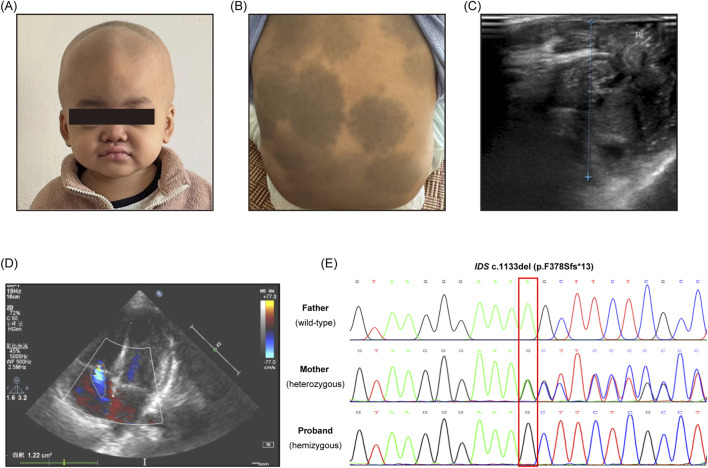
Clinical phenotype, Imaging findings, and Sanger sequencing result of the patient. **(A)** Dysmorphic facial features; **(B)** Mongolian spot; **(C)** Inguinal hernia; **(D)** Mitral and tricuspid regurgitation; **(E)** Sanger sequencing validation of the IDS frameshift variant in the proband and parents (The red box indicates the deletion position).

At 1 year of age, a special facial appearance gradually emerged (Figure 1A), with multiple Mongolian spots (Figure 1B), inguinal hernia (Figure 1C), and mild regurgitation of the mitral and tricuspid valves (Figure 1D). Non-contrast CT scan of the cervical and thoracic spine showed no significant abnormalities. Non-contrast MR angiography of the cerebral arteries showed absence of the left posterior communicating artery, with no other anomalies observed in the remaining cerebral arteries. No abnormalities were identified in the liver, gallbladder, pancreas, or spleen.

### 3.2 Genetic sequencing results

Newborn genetic screening indicated a single-nucleotide deletion mutation in the *IDS* gene of the patient. The variant was located at ChrX: 148,568,503 delA, corresponding to NM_000202.8(*IDS*):c.1133delA (p.Phe378SerfsTer13). This frameshift mutation occurs in exon eight of the *IDS* gene and results in a shift of the reading frame starting at phenylalanine 378, generating 13 aberrant amino acids followed by a premature termination codon, leading to truncation of the iduronate-2-sulfatase protein.

Sanger sequencing chromatograms confirmed the presence and inheritance pattern of the *IDS* gene c.1133delA variant (Figure 1E). At the deletion site, the proband exhibited a single shifted peak pattern consistent with a hemizygous deletion on the X chromosome. The mother showed overlapping wild-type and mutant peaks at the same position, indicating heterozygosity, whereas the father displayed a single wild-type peak, confirming the absence of the variant. These results are consistent with maternal inheritance of the *IDS* frameshift mutation.

### 3.3 Model construction and conformational analysis

According to previous reports (Froissart et al., 1995; Villani et al., 2000; Demydchuk et al., 2017), IDS proteins typically exist or function as dimers. During the secretion process of the IDS enzyme, the first 33 amino acid residues (signal peptide) are cleaved. Each IDS monomer consists of two domains: an SD1 domain (residues 34–443) and an SD2 domain (455–550) with an overall α/β topology, featuring 10 α-helices, 2 β-sheets, and 1 loop region. The active site of IDS is located in a cleft containing many basic residues, and the interaction between SD1 and SD2 domains gives the protein a positive surface potential, consistent with its binding to negatively charged polymer substrates.

**FIGURE 2:**
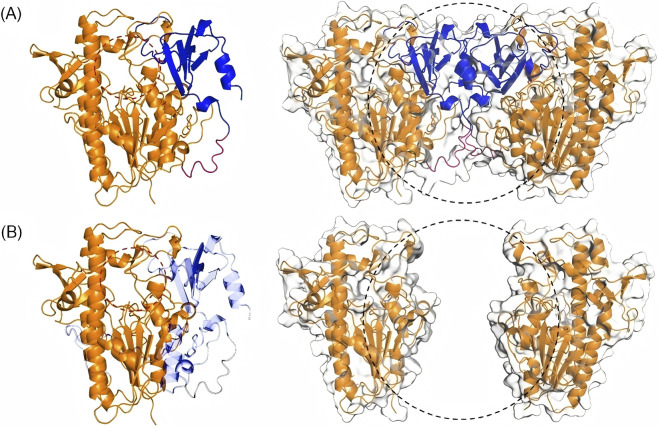
3D Topological Structures of Wild type and Mutant IDS Proteins. **(A)** The wild type Iduronate-2-sulfatase structure, with the SD1 domain labeled in orange, the SD2 domain in blue, the loop region in red, and the ligand active pocket position marked with a red circle, and the structural change site in Iduronate-2-sulfatase marked with a black circle; **(B)** The mutant Iduronate-2-sulfatase structure, with the mutated and missing residues indicated in transparent light blue.

The c.1133delA mutation in the *IDS* gene results in a nucleotide deletion, a frameshift mutation, altering the amino acid sequence from residues p.F378_389, and introducing a premature stop codon at position 390, thereby leading to premature termination of protein translation. The mutation site is located in the SD1 domain. This premature termination leads to the complete absence of a loop region and the SD2 domain of the enzyme (Figure 2). This structural region is crucial for maintaining the stability of the dimeric structure. It contributes to the partial loss of the ligand-binding pocket, disrupting the protein’s positive surface charge potential. Consistent with this structural disruption, pathogenicity assessment using MutPred-LOF, a loss-of-function–oriented predictor for frameshift and stop-gain variants, yielded a score of 0.638, exceeding the recommended threshold of 0.5. This result supports a high likelihood that the c.1133delA variant leads to functional loss through truncation of essential structural domains rather than subtle local perturbations.

### 3.4 Protein-ligand interaction prediction

**FIGURE 3:**
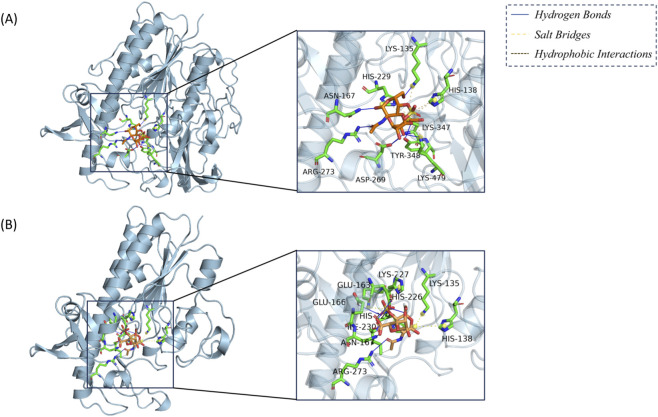
Interaction Analysis of Wild type and Mutant IDS Protein-Ligand Complexes. **(A)** Wild type; **(B)** Mutant; ligand is marked in orange, interacting residues of the protein are marked in green.

Due to the proximity of the mutation site to the protein–ligand binding pocket, molecular docking was performed to evaluate its impact on protein–ligand interactions. Following the binding mode reported by Demydchuk (Demydchuk et al., 2017), derma-tan sulfate was selected as the ligand and docked to both wild-type and mutant IDS protein structures. The protein ligand complex conformations with the lowest binding free energy (top-ranked pose) were selected for subsequent analysis. Residue-level interaction patterns were further analyzed using the PLIP online server (Figure 3; Supplementary Tables S1 and S2).

PLIP analysis (Supplementary Tables S1–S2) revealed a marked reorganization of residue-level contacts between the wild-type and mutant IDS–ligand complexes. In the wild-type structure, seven short hydrogen bonds involving Lys135, Asn167, Asp269, Arg273, His348, and Lys479, together with five Lys/His–carboxylate salt bridges (e.g., Lys135, His138, His229, Lys347, Lys479), form a dense charge-driven interaction network that stabilizes the carboxylate groups of dermatan sulfate. In contrast, the mutant protein retains only five hydrogen bonds, including newly formed contacts with Glu163 and Glu166 that are generally longer and weaker, as well as a single weak hydrophobic contact with Ile230. Several key stabilizing hydrogen bonds present in the wild-type protein, particularly those involving Lys135, Asp269, His348, and Lys479, are weakened or lost in the mutant structure, whereas interactions involving Asn167 and Arg273 are only partially preserved and display altered geometries with increased distances. In addition, the salt-bridge pattern is reorganized: the Lys135/His138 network is weakened, and new salt bridges mediated by His226 and Lys227 now engage ligand sulfate groups rather than the original carboxylate clusters, indicating a shift in electrostatic anchoring sites within the binding pocket.

Consistent with these structural observations, docking calculations further indicated a reduction in binding affinity, with the wild-type IDS–ligand complex exhibiting a binding free energy of −3.62 kcal/mol compared with −3.27 kcal/mol for the mutant. Although the absolute energy difference is modest, it aligns with the observed loss and rearrangement of key hydrogen bonds and salt bridges, collectively supporting a weakened and less optimal ligand-binding mode in the mutant protein.

### 3.5 Genome-scale metabolic model analysis

FBA analysis revealed that *IDS* knockout had no impact on cellular growth capacity (Table 1). Both the wild-type and knockout models achieved identical maximum biomass flux of 124.87. This result indicates that *IDS* is not essential for cellular growth under the modeled conditions, consistent with GAG degradation representing a catabolic process not directly required for biomass synthesis.

**TABLE 1:** Effect of IDS knockout on cellular growth rate.

Model	Maximum biomass flux	Relative change
Wild-type (WT)	124.87	—
*IDS* knockout (KO)	124.87	0%

### 3.6 GAG degradation capacity exhibits a trade-off relationship with cellular growth

**FIGURE 4:**
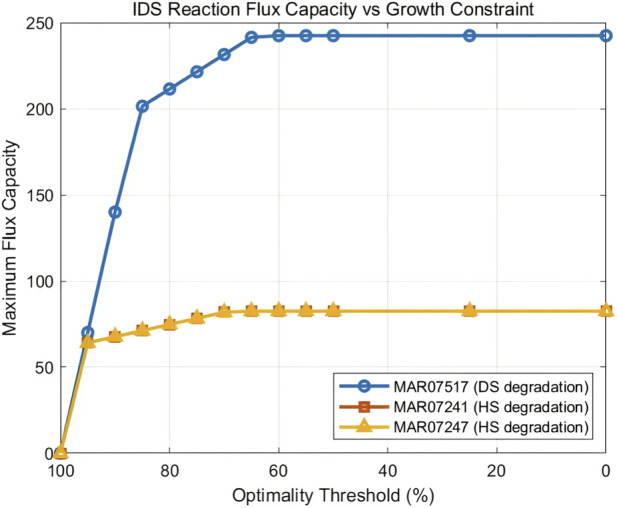
Relationship between IDS reaction flux capacity and growth constraint. Maximum achievable flux for the three *IDS*-associated reactions at optimality thresholds ranging from 0% to 100%. All three reactions show zero flux at 100% threshold and increas-ing capacity as the growth constraint is relaxed, following a saturation curve pattern. MAR07517 (dermatan sulfate degradation) shows approximately 3-fold higher maximum capacity than MAR07241 and MAR07247 (heparan sulfate degradation).

FVA analysis of the three *IDS*-associated reactions at different optimality thresholds revealed a saturation-like relationship between GAG degradation capacity and growth constraint relaxation (Figure 4; Table 2). At the 100% threshold, all three reactions showed zero maximum flux, indicating that optimal cellular growth can be achieved without engagement of GAG degradation pathways. As the growth constraint was relaxed, flux capacity increased rapidly between 100% and 85% thresholds, then gradually approached saturation below 60% threshold.

**TABLE 2:** Maximum flux capacity of IDS-associated reactions at different FVA optimality thresholds.

Optimality threshold (%)	MAR07517 (DS)	MAR07241 (HS)	MAR07247 (HS)
100	0	0	0
90	140.00	67.68	67.68
75	221.43	78.28	78.28
50	242.42	82.47	82.47

DS, dermatan sulfate; HS, heparan sulfate.

### 3.7 IDS knockout affects a highly localized set of reactions

**FIGURE 5:**
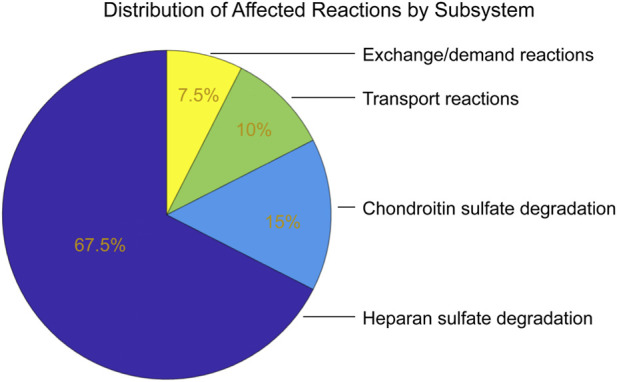
Subsystem distribution of affected reactions following IDS knockout. All 40 significantly affected reactions are confined to glycosaminoglycan degradation-related pathways. No reactions were affected in central carbon metabolism, amino acid metabolism, nucleotide metabolism, lipid metabolism, or any other metabolic subsystems.

Genome-wide FVA at the 90% optimality threshold identified 42 reactions with significantly altered flux ranges (|Δrange| > 1). After excluding two reactions attributed to numerical noise, 40 reactions showed genuine metabolic perturbation. All affected reactions were confined to glycosaminoglycan degradation-related pathways (Figure 5), including 27 reactions in heparan sulfate degradation, 6 in chondroitin sulfate degradation (which includes dermatan sulfate), 4 in lysosomal transport, and 3 in exchange/demand reactions.

No reactions were affected in central carbon metabolism, amino acid metabolism, nucleotide metabolism, lipid metabolism, oxidative phosphorylation, or other metabolic subsystems outside glycosaminoglycan degradation, despite the model encompassing 12,971 reactions across the human metabolic network.

### 3.8 Complete loss of GAG degradation capacity following IDS knockout

The three IDS-associated reactions showed complete loss of flux capacity following knockout (Table 3). At the 90% threshold, the flux range of MAR07517 changed from [0, 140.00] in wild-type to [0, 0] in knockout, representing 100% loss of dermatan sulfate degradation capacity. Similarly, MAR07241 and MAR07247 flux ranges changed from [0, 67.68] to [0, 0], representing 100% loss of heparan sulfate degradation capacity.

**TABLE 3:** Flux range changes of IDS-associated reactions following knockout (90% optimality threshold).

Reaction ID	Subsystem	WT flux range	KO flux range	Capacity loss
MAR07517	CS degradation	[0, 140.00]	[0, 0]	100%
MAR07241	HS degradation	[0, 67.68]	[0, 0]	100%
MAR07247	HS degradation	[0, 67.68]	[0, 0]	100%

### 3.9 Robustness of findings across FVA parameters

Sensitivity analysis confirmed the robustness of principal findings across different optimality thresholds (Table 4). At both 75% and 50% thresholds, the same 40 reactions were identified as significantly affected, with identical subsystem distribution.

**TABLE 4:** Sensitivity analysis: affected reactions at different FVA optimality thresholds.

Optimality threshold (%)	Affected reactions	Subsystem distribution
90	40	All in GAG degradation
75	40	All in GAG degradation
50	40	All in GAG degradation

### 3.10 Identification of potentially accumulating metabolites

Reporter metabolite analysis identified 62 metabolites associated with the 40 affected reactions, of which 34 were classified as potentially accumulating (Table 5). These comprised 27 heparan sulfate degradation intermediates, 5 dermatan sulfate degradation intermediates, and 2 forms of L-iduronic acid (cytosolic and extracellular). Notably, 32 of 34 potentially accumulating metabolites (94%) localize to the lysosome, consistent with MPS II classification as a lysosomal storage disorder.

**TABLE 5:** Classification of potentially accumulating metabolites following IDS knockout.

Category	Count	Compartment
Heparan sulfate degradation intermediates	27	Lysosome
Dermatan sulfate degradation intermediates	5	Lysosome
L-iduronic acid	2	Cytosol, extracellular

## 4 Discussion

In this study, we combined clinical characterization, structural modeling, enzyme activity assays, and genome-scale metabolic modeling to investigate the pathogenic mechanism of a novel *IDS* frameshift mutation (NM_000202.8:c.1133delA; p.Phe378SerfsTer13). Structural analysis demonstrated that this mutation truncates the Iduronate-2-sulfatase protein, abolishes the SD2 domain, disrupts dimer stability, and impairs the ligand-binding pocket, consistent with the markedly reduced enzyme activity observed in the patient sample.

Genome-scale metabolic modeling was employed to systematically assess the metabolic consequences of IDS deficiency. The principal finding is that IDS knockout results in a highly localized but complete metabolic defect. Despite the Human-GEM model encompassing 12,971 reactions spanning all major human metabolic pathways, only 40 reactions (0.31%) exhibited significant flux range changes, all strictly confined to glycosaminoglycan degradation pathways. No detectable metabolic perturbations were observed in central carbon metabolism, amino acid metabolism, nucleotide metabolism, lipid metabolism, or oxidative phosphorylation.

The observation that *IDS* knockout has no impact on maximum biomass flux is mechanistically informative. It indicates that glycosaminoglycan degradation operates inde-pendently of the core metabolic network required for cellular growth and proliferation. This metabolic independence provides a mechanistic explanation for the clinical observation that patients with MPS II can survive for decades despite complete loss of IDS activity (Muenzer et al., 2006).

Importantly, the absence of global metabolic reprogramming in the model does not contradict the multisystemic clinical manifestations of MPS II. Lysosomal storage disorders are characterized by progressive, long-term accumulation of undegraded substrates rather than acute disruption of global metabolic fluxes (Neufeld and Muenzer, 2019). The complete loss of glycosaminoglycan degradation capacity predicted here leads to persistent lysosomal accumulation of heparan sulfate and dermatan sulfate intermediates, which over time impairs lysosomal function, disrupts vesicular trafficking and autophagy, and induces secondary cellular stress responses (Platt et al., 2018). These cumulative and indirect effects are amplified in tissues with high extracellular matrix turnover or limited regenerative capacity, including the central nervous system, heart valves, liver, spleen, and skeletal system, thereby giving rise to the systemic phenotype of MPS II (D’Avanzo et al., 2020).

The highly localized nature of the metabolic defect identified in this study provides network-level support for the therapeutic rationale of enzyme replacement therapy (ERT). Restoration of a single lysosomal enzyme is mechanistically sufficient to correct the underlying metabolic blockade, consistent with clinical evidence that idursulfase treatment reduces urinary glycosaminoglycan levels and hepatosplenomegaly in MPS II patients (Muenzer et al., 2006; Muenzer et al., 2007), for gene knockout studies although neurological manifestations remain challenging due to limited blood–brain barrier penetration. Beyond supporting the rationale for enzyme replacement, the IDS-deficient Human-GEM model also highlights a small, highly localized set of reactions within heparan sulfate and dermatan sulfate degradation as network-level metabolic vulnerabilities. Although the present study does not perform compound screening, these pathway- and reaction-level nodes could serve as a starting point for future target prioritization and therapeutic design. In this context, multi-scale *in silico* frameworks that integrate gene-level perturbations with protein–ligand interaction prediction and virtual drug screening, such as the approach described by Nayak and Mallick (Nayak and Mallick, 2024), provide a conceptual template for extending our findings toward systematic drug repurposing in MPS II.

From a methodological perspective, this work highlights the importance of flux variability analysis for reliable gene knockout simulations in genome-scale metabolic models (Mahadevan and Schilling, 2003). Preliminary flux balance analysis alone suggested widespread metabolic changes following IDS knockout, whereas flux variability analysis revealed these apparent changes to be artifacts of alternative optimal solutions. This underscores the necessity of appropriate constraint-based methods when interpreting genome-scale modeling results in the context of inherited metabolic diseases.

Several limitations should be acknowledged. Genome-scale modeling captures metabolic capabilities under steady-state assumptions and does not explicitly model kinetic parameters, temporal substrate accumulation, or tissue-specific metabolism. Future studies integrating tissue-resolved models, longitudinal clinical data, and multi-omics measurements will be required to further refine the systems-level understanding of MPS II pathogenesis.

## 5 Conclusion

In conclusion, genome-scale metabolic modeling of Iduronate-2-sulfatase deficiency reveals a highly localized but complete metabolic blockade confined to glycosaminoglycan degradation pathways. In a human metabolic network comprising 12,971 reactions, only 40 reactions (0.31%) were significantly affected, all within heparan sulfate and dermatan sulfate degradation. Importantly, IDS knockout has no impact on cellular growth capacity, demonstrating that glycosaminoglycan degradation operates independently of core biosynthetic metabolism.

These findings provide mechanistic insight into the pathogenesis of MPS II and support the clinical efficacy of enzyme replacement therapy, while also highlighting the utility of genome-scale metabolic modeling for dissecting the molecular basis of inherited lysosomal storage disorders. Validation in additional patients and integration with tissue-specific and longitudinal data will further strengthen these conclusions.

## Data Availability

The data presented in this study are deposited in the OMIX repository, accession number OMIX008658 (https://ngdc.cncb.ac.cn/omix/).
