# Supplementary material for: Revealing the role of a novel IDS gene mutation in mucpolysaccharidosis type II: insights from computational analysis
Source: Front Mol Biosci. 2026 Apr 2;13:1734111. doi: 10.3389/fmolb.2026.1734111 (PMC13084169; doi:10.3389/fmolb.2026.1734111)
Supplement: Supplementary file 1 [file Supplementaryfile1.docx]

**Table S1 Wild-type IDS–ligand interactions**

| **Interaction type** | **Residue (Chain)** | **Amino acid** | **Distance (Å)** | **Angle (°)** | **Ligand atom(s)** | **Notes** |
| --- | --- | --- | --- | --- | --- | --- |
| Hydrogen bond | 135B | Lys | 1.97–2.74 | 130.27 | O3 | Stable H-bond |
| Hydrogen bond | 167B | Asn | 1.98–2.94 | 154.16 | O3 | Stable H-bond |
| Hydrogen bond | 269B | Asp | 1.80–2.64 | 142.19 | O.co2 | Ligand carboxylate |
| Hydrogen bond | 269B | Asp | 2.00–2.91 | 154.58 | O.co2 | Second contact |
| Hydrogen bond | 273B | Arg | 2.24–2.99 | 128.72 | O2 | Electrostatic component |
| Hydrogen bond | 348B | His | 2.60–3.35 | 136.65 | O.co2 | Moderate strength |
| Hydrogen bond | 479B | Lys | 1.75–2.77 | 174.63 | O3 | Strong linear H-bond |
| Salt bridge | 135B | Lys | 5.46 | – | Carboxylate (17,18) | Long-range ionic |
| Salt bridge | 138B | His | 5.07 | – | Carboxylate (17,18) | Ionic interaction |
| Salt bridge | 229B | His | 4.35–5.45 | – | Carboxylate (16,30) | Multiple contacts |
| Salt bridge | 347B | Lys | 3.74 | – | Carboxylate (19,20) | Strong salt bridge |
| Salt bridge | 479B | Lys | 3.04–3.95 | – | Carboxylate (17,18) | Key stabilizing contact |
