# Supplementary material for: Revealing the role of a novel IDS gene mutation in mucpolysaccharidosis type II: insights from computational analysis
Source: Front Mol Biosci. 2026 Apr 2;13:1734111. doi: 10.3389/fmolb.2026.1734111 (PMC13084169; doi:10.3389/fmolb.2026.1734111)
Supplement: Supplementary file 2 [file Supplementaryfile5.docx]

**Table S5 All genes included in the newborn genetic screening panel.**

| **序号** | **Gene** | **HGMD transcript** |
| --- | --- | --- |
| **1** | *ABCC2* | NM_000392.4 |
| **2** | *ABCD1* | NM_000033.3 |
| **3** | *ABCD4* | NM_005050.3 |
| **4** | *ABCG5* | NM_022436.2 |
| **5** | *ABCG8* | NM_022437.2 |
| **6** | *ACAD8* | NM_014384.2 |
| **7** | *ACADM* | NM_000016.5 |
| **8** | *ACADS* | NM_000017.3 |
| **9** | *ACADSB* | NM_001609.3 |
| **10** | *ACADVL* | NM_000018.2 |
| **11** | *ACAT1* | NM_000019.4 |
| **12** | *ACSF3* | NM_174917.3 |
| **13** | *ADA* | NM_000022.3 |
| **14** | *AGL* | NM_000642.2 |
| **15** | *ALDH7A1* | NM_001182.4 |
| **16** | *ALDOB* | NM_000035.3 |
| **17** | *APOB* | NM_000384.2 |
| **18** | *ARG1* | NM_000045.2 |
| **19** | *ARSA* | NM_000487.5 |
| **20** | *ARSB* | NM_000046.4 |
| **21** | *ASL* | NM_000048.3 |
| **22** | *ASS1* | NM_000050.4 |
| **23** | *ATP7A* | NM_000052.6 |
| **24** | *ATP7B* | NM_000053.3 |
| **25** | *BCKDHA* | NM_000709.3 |
| **26** | *BCKDHB* | NM_183050.3 |
| **27** | *BTD* | NM_000060.4 |
| **28** | *BTK* | NM_000061.2 |
| **29** | *CAPN3* | NM_000070.2 |
| **30** | *CBS* | NM_000071.2 |
| **31** | *CFTR* | NM_000492.3 |
| **32** | *COL1A1* | NM_000088.3 |
| **33** | *COL1A2* | NM_000089.3 |
| **34** | *COL2A1* | NM_001844.4 |
| **35** | *CPS1* | NM_001875.4 |
| **36** | *CPT1A* | NM_001876.3 |
| **37** | *CPT2* | NM_000098.2 |
| **38** | *CYBB* | NM_000397.3 |
| **39** | *DBT* | NM_001918.3 |
| **40** | *DDC* | NM_000790.3 |
| **41** | *DSG2* | NM_001943.4 |
| **42** | *DSP* | NM_004415.3 |
| **43** | *DUOX2* | NM_014080.4 |
| **44** | *DYSF* | NM_003494.3 |
| **45** | *ETFA* | NM_000126.3 |
| **46** | *ETFDH* | NM_004453.3 |
| **47** | *ETHE1* | NM_014297.4 |
| **48** | *F9* | NM_000133.3 |
| **49** | *FAH* | NM_000137.2 |
| **50** | *FBN1* | NM_000138.4 |
| **51** | *FGFR3* | NM_000142.4 |
| **52** | *G6PC* | NM_000151.3 |
| **53** | *G6PD* | NM_001042351.2 |
| **54** | *GAA* | NM_000152.4 |
| **55** | *GALC* | NM_000153.3 |
| **56** | *GALE* | NM_000403.3 |
| **57** | *GALK1* | NM_000154.1 |
| **58** | *GALNS* | NM_000512.4 |
| **59** | *GALT* | NM_000155.3 |
| **60** | *GAMT* | NM_000156.5 |
| **61** | *GBA* | NM_001005741.2 |
| **62** | *GCDH* | NM_000159.3 |
| **63** | *GCH1* | NM_000161.2 |
| **64** | *GJB2* | NM_004004.5 |
| **65** | *GJB3* | NM_024009.2 |
| **66** | *GLA* | NM_000169.2 |
| **67** | *GLB1* | NM_000404.3 |
| **68** | *GLDC* | NM_000170.2 |
| **69** | *GNPTAB* | NM_024312.4 |
| **70** | *GUSB* | NM_000181.3 |
| **71** | *HADHA* | NM_000182.4 |
| **72** | *HADHB* | NM_000183.2 |
| **73** | *HBB* | NM_000518.4 |
| **74** | *HEXA* | NM_000520.5 |
| **75** | *HLCS* | NM_000411.7 |
| **76** | *HMGCL* | NM_000191.2 |
| **77** | *HPD* | NM_002150.2 |
| **78** | *IDS* | NM_000202.7 |
| **79** | *IDUA* | NM_000203.4 |
| **80** | *IL10RA* | NM_001558.3 |
| **81** | *IL2RG* | NM_000206.2 |
| **82** | *IVD* | NM_002225.3 |
| **83** | *JAG1* | NM_000214.2 |
| **84** | *KCNH2* | NM_000238.3 |
| **85** | *KCNQ1* | NM_000218.2 |
| **86** | *KCNQ2* | NM_172107.3 |
| **87** | *L2HGDH* | NM_024884.2 |
| **88** | *LAMA2* | NM_000426.3 |
| **89** | *LDLR* | NM_000527.4 |
| **90** | *MAT1A* | NM_000429.2 |
| **91** | *MCCC1* | NM_020166.4 |
| **92** | *MCCC2* | NM_022132.4 |
| **93** | *MECP2* | NM_004992.3 |
| **94** | *MMAA* | NM_172250.2 |
| **95** | *MMAB* | NM_052845.3 |
| **96** | *MMACHC* | NM_015506.2 |
| **97** | *MMUT* | NM_000255.3 |
| **98** | *MTHFR* | NM_005957.4 |
| **99** | *MTR* | NM_000254.2 |
| **100** | *MT-RNR1* |  |
| **101** | *MTRR* | NM_002454.2 |
| **102** | *MT-TL1* |  |
| **103** | *MYBPC3* | NM_000256.3 |
| **104** | *MYH7* | NM_000257.3 |
| **105** | *MYO5B* | NM_001080467.2 |
| **106** | *NAGS* | NM_153006.2 |
| **107** | *NF1* | NM_000267.3 |
| **108** | *NF2* | NM_000268.3 |
| **109** | *NOTCH2* | NM_024408.3 |
| **110** | *NPC1* | NM_000271.4 |
| **111** | *NPC2* | NM_006432.3 |
| **112** | *NPHS1* | NM_004646.3 |
| **113** | *OCA2* | NM_000275.2 |
| **114** | *OTC* | NM_000531.5 |
| **115** | *PAH* | NM_000277.2 |
| **116** | *PC* | NM_000920.3 |
| **117** | *PCCA* | NM_000282.3 |
| **118** | *PCCB* | NM_000532.4 |
| **119** | *PCSK9* | NM_174936.3 |
| **120** | *PHKA2* | NM_000292.2 |
| **121** | *PKLR* | NM_000298.6 |
| **122** | *PKP2* | NM_004572.3 |
| **123** | *PNPO* | NM_018129.3 |
| **124** | *PRODH* | NM_016335.4 |
| **125** | *PRRT2* | NM_145239.2 |
| **126** | *PTPN11* | NM_002834.4 |
| **127** | *PTS* | NM_000317.2 |
| **128** | *PYGL* | NM_002863.4 |
| **129** | *QDPR* | NM_000320.2 |
| **130** | *RAF1* | NM_002880.3 |
| **131** | *RB1* | NM_000321.2 |
| **132** | *RYR2* | NM_001035.2 |
| **133** | *SCN1A* | NM_001165963.2 |
| **134** | *SCN2A* | NM_021007.2 |
| **135** | *SCN5A* | NM_198056.2 |
| **136** | *SGSH* | NM_000199.4 |
| **137** | *SLC10A1* | NM_003049.3 |
| **138** | *SLC12A3* | NM_000339.2 |
| **139** | *SLC22A5* | NM_003060.3 |
| **140** | *SLC25A13* | NM_014251.2 |
| **141** | *SLC25A15* | NM_014252.3 |
| **142** | *SLC25A20* | NM_000387.5 |
| **143** | *SLC26A4* | NM_000441.1 |
| **144** | *SLC37A4* | NM_001164277.1 |
| **145** | *SLC6A8* | NM_005629.3 |
| **146** | *SMPD1* | NM_000543.4 |
| **147** | *SOS1* | NM_005633.3 |
| **148** | *SRD5A2* | NM_000348.3 |
| **149** | *SUCLG1* | NM_003849.3 |
| **150** | *TAT* | NM_000353.2 |
| **151** | *TG* | NM_003235.4 |
| **152** | *TH* | NM_199292.2 |
| **153** | *TNNI3* | NM_000363.4 |
| **154** | *TNNT2* | NM_001001430.2 |
| **155** | *TSC1* | NM_000368.4 |
| **156** | *TSC2* | NM_000548.4 |
| **157** | *TSHR* | NM_000369.2 |
| **158** | *TYR* | NM_000372.4 |
| **159** | *UGT1A1* | NM_000463.2 |
| **160** | *USH2A* | NM_206933.2 |
