# Supplementary material for: Revealing the role of a novel IDS gene mutation in mucpolysaccharidosis type II: insights from computational analysis
Source: Front Mol Biosci. 2026 Apr 2;13:1734111. doi: 10.3389/fmolb.2026.1734111 (PMC13084169; doi:10.3389/fmolb.2026.1734111)
Supplement: Supplementary file 4 [file Supplementaryfile2.docx]

**Table S2 Mutant-type IDS–ligand interactions**

| **Interaction type** | **Residue (Chain)** | **Amino acid** | **Distance (Å)** | **Angle (°)** | **Ligand atom(s)** | **Notes** |
| --- | --- | --- | --- | --- | --- | --- |
| Hydrophobic | 230A | Ile | 3.53 | – | C14 | Weak hydrophobic contact |
| Hydrogen bond | 163A | Glu | 3.08–3.97 | 157.61 | O3 | Longer, weaker H-bond |
| Hydrogen bond | 163A | Glu | 2.11–2.98 | 149.14 | O3 | Moderate stability |
| Hydrogen bond | 166A | Glu | 3.42–4.09 | 128.12 | O.co2 | Weak interaction |
| Hydrogen bond | 167A | Asn | 2.08–3.06 | 162.18 | O.co2 | Preserved contact |
| Hydrogen bond | 273A | Arg | 2.69–3.40 | 126.89 | O2 | Retained ionic feature |
| Salt bridge | 135A | Lys | 3.35 | – | Carboxylate (16,30) | Maintained |
| Salt bridge | 138A | His | 4.73 | – | Carboxylate (16,30) | Weakened |
| Salt bridge | 226A | His | 4.33 | – | Sulfate (31) | New interaction |
| Salt bridge | 227A | Lys | 3.50 | – | Sulfate (31) | New interaction |
