# Supplementary material for: Revealing the role of a novel IDS gene mutation in mucpolysaccharidosis type II: insights from computational analysis
Source: Front Mol Biosci. 2026 Apr 2;13:1734111. doi: 10.3389/fmolb.2026.1734111 (PMC13084169; doi:10.3389/fmolb.2026.1734111)
Supplement: Supplementary file 5 [file Supplementaryfile4.docx]

**S4 IDS Mutant sequence**

MPPPRTGRGLLWLGLVLSSVCVALGSETQANSTTDALNVLLIIVDDLRPSLGCYGDKLVRSPNIDQLASHSLLFQNAFAQQAVCAPSRVSFLTGRRPDTTRLYDFNSYWRVHAGNFSTIPQYFKENGYVTMSVGKVFHPGISSNHTDDSPYSWSFPPYHPSSEKYENTKTCRGPDGELHANLLCPVDVLDVPEGTLPDKQSTEQAIQLLEKMKTSASPFFLAVGYHKPHIPFRYPKEFQKLYPLENITLAPDPEVPDGLPPVAYNPWMDIRQREDVQALNISVPYGPIPVDFQRKIRQSYFASVSYLDTQVGRLLSALDDLQLANSTIIAFTSDHGWALGEHGEWAKYSNFDVATHVPLIFYVPGRTASLPEAGEKLSLTSTLLIPPHS
